# Native-lung complications following single-lung transplantation for interstitial lung disease: an in-depth analysis

**DOI:** 10.1186/s12890-024-03009-6

**Published:** 2024-04-24

**Authors:** Toshikazu Watanabe, Takashi Hirama, Ken Onodera, Hirotsugu Notsuda, Hisashi Oishi, Hiromichi Niikawa, Kazuyoshi Imaizumi, Yoshinori Okada

**Affiliations:** 1 Department of Thoracic Surgery, Institute of Development, Aging and Cancer, Tohoku University, Sendai, Miyagi Japan; 2 Division of Organ Transplantation, Tohoku University Hospital, Sendai, Miyagi Japan; 3 Department of Respiratory Medicine, Fujita Health University School of Medicine, Toyoake, Aichi Japan

**Keywords:** Lung transplant, Single lung transplant, Native lung, Pneumothorax, Aspergillus, Acute exacerbation

## Abstract

**Background:**

Interstitial lung disease (ILD) represents a heterogeneous group of lung disorders characterized by fibrotic lung tissue changes. In regions with severe donor shortages, single-lung transplantation (SLTx) is often preferred over bilateral lung transplantation for advanced ILD. However, temporal changes and complications in the retained native lung remain poorly understood.

**Methods:**

A retrospective analysis of 149 recipients who had undergone SLTx was conducted, including 34 ILD SLTx recipients. Native-lung volume, radiological alterations, and perfusion were assessed at distinct post-SLTx time points. Statistical analyses compared ILD and non-ILD SLTx groups.

**Results:**

Our study revealed a progressive reduction in native-lung volume over time, accompanied by radiographic deterioration and declining perfusion. Complications in the retained native lung were observed, such as pneumothorax (29.4%), pulmonary aspergillosis (11.8%), and acute exacerbation (8.9%). Long-term survival rates were similar between ILD and non-ILD SLTx recipients.

**Conclusions:**

This study illuminates the unique challenges and complications with respect to the native lung following SLTx for ILD. Ongoing monitoring and tailored management are essential. Despite limitations, this research contributes to our understanding of the temporal progression of native-lung complications post-SLTx for ILD, underscoring the need for further investigation.

**Supplementary Information:**

The online version contains supplementary material available at 10.1186/s12890-024-03009-6.

## Background

In performing lung transplantation (LTx) in regions with severe donor shortages, such as Japan, single-lung transplantation (SLTx) is often preferred over bilateral lung transplantation (BLTx) because of the critical need for available organs [1–3]. This approach is particularly relevant in cases of advanced interstitial lung disease (ILD) where conventional treatments have proven ineffective [4, 5]. However, the unique aspect of SLTx in ILD lies in the fact that one native lung is retained alongside the transplanted lung, making recipients susceptible to complications that can significantly impact their health and long-term outcomes. While existing research has explored LTx outcomes for ILD, there remains a crucial gap in our understanding of the temporal progression of complications in the native lung, particularly fibrosis [6, 7]. This study seeks to address this need for a comprehensive understanding of SLTx in ILD recipients. By analyzing the temporal progression of native-lung complications and their influence on recipient outcomes, we aim to provide valuable insights into this ongoing process. Our examination will encompass changes in native-lung volume, radiological alterations, and perfusion in the native lung, shedding light on the persistent nature of ILD following SLTx.

## Methods

### Sample collection

A retrospective analysis was conducted on 149 recipients who had undergone deceased-donor LTx at Tohoku University Hospital (TUH) between January 2000 and March 2023, with follow-up extending to June 2023. Among this cohort, 34 recipients who had undergone SLTx for ILD (ILD SLTx) were selected for the study, with an additional 54 who had undergone SLTx for conditions other than ILD (non-ILD SLTx) included as a control group. The analysis excluded recipients who had undergone BLTx (*n* = 61). It is worth noting that prior studies have documented the transplant registry in Japan [8], intraoperative management, and chronic phase care [9, 10], immunosuppressive therapy [11], histocompatibility testing after LTx [12], and antimicrobial prophylaxis [9, 13]. The diagnosis of acute exacerbation of ILD in the native lung following SLTx was established on the basis of the emergence of new ground-glass opacities exclusively within the native lung post-discharge from the intensive care unit (ICU), explicitly excluding cases associated with heart failure [14].

### Quantitative assessment of native-lung volume

**Fig. 1:**
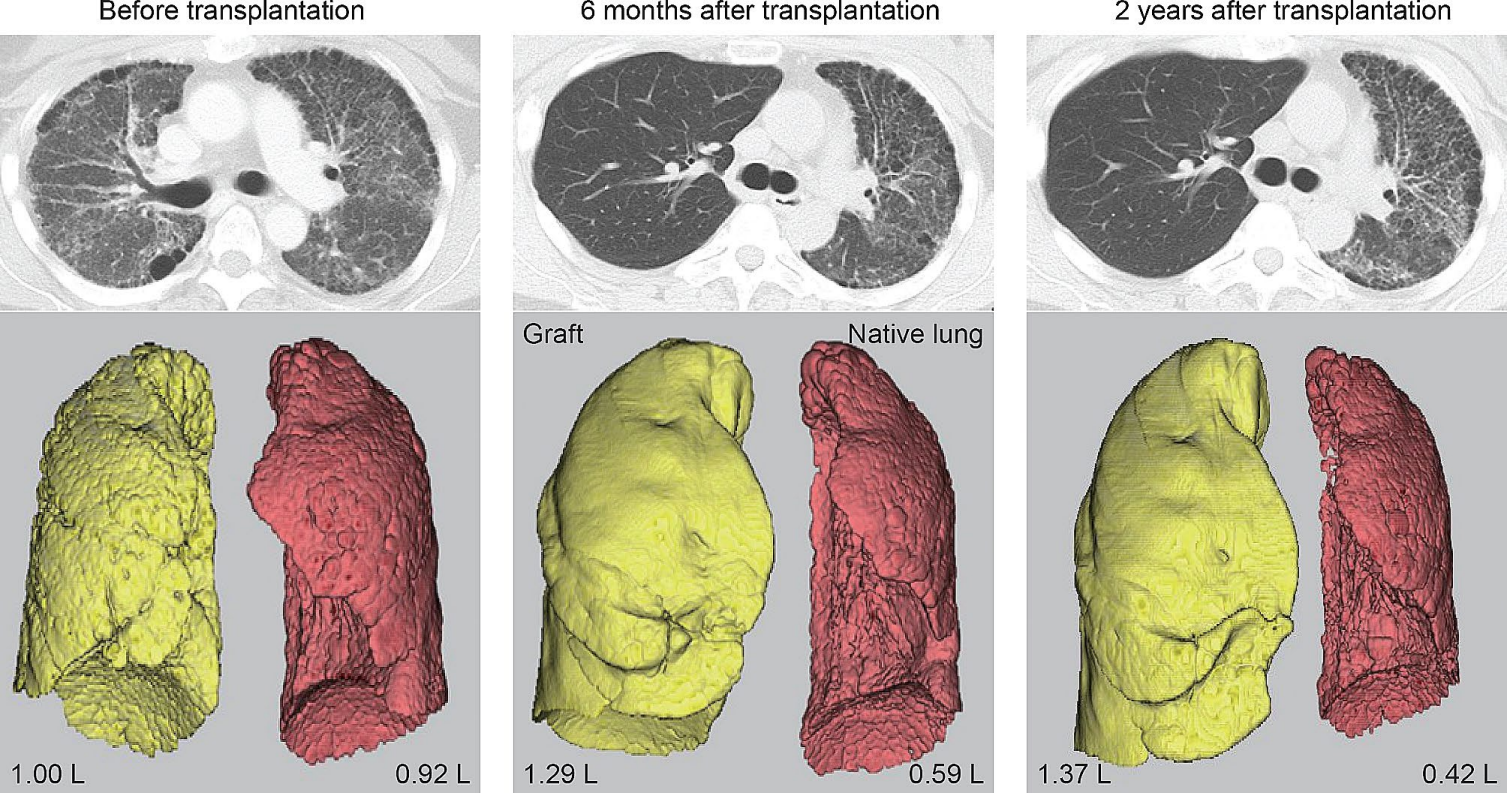
Representative images of three-dimensional lung reconstructions generated using SYNAPSE VINCENT and used to assess the native-lung volume in recipients who had undergone single-lung transplantation at three distinct time points

Lung perfusion scintigraphy is routinely conducted at our transplant center, performed at the time of listing for LTx, as well as at 3 and 6 months post-transplant, and subsequently on an annual basis. Native-lung status was evaluated through thin-slice computed tomography (CT) scans at three distinct time points: pre-SLTx, six months post-SLTx, and two years post-SLTx for recipients who survived that far. These CT data were utilized to generate three-dimensional (3D) lung reconstructions. Lung volume measurements were derived using three-dimensional reconstruction software (SYNAPSE VINCENT; Fujifilm Co., Ltd., Tokyo, Japan). In brief, 3D lung images were rendered by isolating lung and bronchial images from the CT data, followed by bronchial component removal. Subsequently, the software autonomously calculated the volumetric measurements of the reconstructed lungs (Fig. 1).

### Radiological assessment of native-lung alterations

CT images were independently analyzed by at least two experts specializing in respiratory medicine, in collaboration with a radiologist. Both native and transplanted lungs underwent evaluation utilizing a semi-quantitative method as proposed by Warrick et al. [15], commonly referred to as the Warrick score. This method involves the streamlined scoring of pulmonary fibrosis from chest CT images and is widely employed in various fields [16–18]. The severity score documented five fundamental lung lesions, including ground-glass opacity, irregularities in pleural margins, septal lines, honeycombing, and subpleural cysts, each rated on a scale of 1 to 5. The cumulative severity score was determined by summing the values for each observed lesion, ranging from 0 (no lesions) to 15 (all lesions present). The extent score was based on the number of lung segments affected by each lesion type, with a score of 1 indicating involvement in one to three segments, 2 in four to nine segments, and 3 in more than nine segments. The extent score had a potential range of 0 to 15, and the overall Warrick score was calculated by combining severity and extent scores, resulting in a total score ranging from 0 to 30, covering the entire spectrum of potential outcomes.

### Native-lung perfusion evaluation

The evaluation of native-lung perfusion involved assessing the right-to-left pulmonary perfusion ratio at the three aforementioned time points. This scintigraphy procedure was performed after intravenous administration of 99mTc, with the recipient in a supine position. A single-head gamma camera detected anterior and posterior counts per view, with the cumulative view used to calculate relative perfusion. The perfusion of the target side was expressed as a percentage of total lung perfusion.

### Statistical analysis

The presentation of variables included medians (interquartile range [IQR]) and actual numbers (percentages). Categorical variables were subjected to the chi-square or Fisher’s exact tests, while continuous variables were analyzed using the Mann-Whitney U test. Time-to-event outcomes were modeled using the Kaplan-Meier method, and group differences were evaluated with the log-rank test. Unadjusted survival analyses were conducted to account for the relatively small sample size and avoid overfitting. Statistical significance was defined as a *p*-value below 0.05. All statistical analyses were performed using EZR (Saitama Medical Center, Jichi Medical University, Saitama, Japan), which is a graphical user interface for R (The R Foundation for Statistical Computing, Vienna, Austria). Essentially, EZR is a modified version of R Commander tailored to accommodate the statistical functions frequently used in biostatistics.

## Results

### Characteristics of recipients who had undergone single-lung transplantation for ILD

During the study, a total of 149 patients underwent deceased-donor LTx at TUH. Among these patients, 34 underwent ILD SLTx, while 54 underwent non-ILD SLTx (Table 1). Within the ILD SLTx group, the most prevalent pulmonary disease was idiopathic pulmonary fibrosis (IPF) (*n* = 14), followed by connective tissue disease (CTD) -associated ILD (*n* = 11). In the non-ILD SLTx group, the most frequently observed condition was lymphangioleiomyomatosis (LAM) (*n* = 35), followed by chronic obstructive pulmonary disease (*n* = 9), and chronic pulmonary graft-versus-host disease (*n* = 4). While ILD SLTx recipients tended to be older than non-ILD SLTx recipients (median age 53 vs. median age 47), this difference did not reach statistical significance. However, the proportion of male recipients in the ILD SLTx group was significantly higher (67.7% vs. 22.2%), and their BMI was also higher (median BMI 22.2 kg/m^2^ vs. median BMI 17.7 kg/m^2^). Regarding the ratio of right-side SLTx, no statistically significant difference was observed between the ILD SLTx group (38.2%) and the non-ILD SLTx group (57.4%) (*p* = 0.125). With respect to perioperative complications, there were no significant disparities in terms of ischemia time, duration of mechanical ventilation, the proportion of recipients requiring tracheostomy, the percentage requiring delayed closure procedure, or the duration of ICU stay between the ILD SLTx and non-ILD SLTx groups. The three-month mortality rate in the ILD SLTx group was 3.9%, and that in the non-ILD SLTx group was 3.7%, with no statistically significant difference detected (*p* = 0.999). In contrast, albeit diverging from the primary focus of this study, supplemental Table 1 presents the characteristics of individuals who underwent bilateral lung transplantation (BLTx) for ILD.

**Table 1:** Characteristics of recipients who had undergone single-lung transplant for ILD or otherwise

Characteristic	Total *n* = 149	ILD SLTx *n* = 34	non-ILD SLTx *n* = 54	*p*-value
Age, years (IQR)	46 (36–53)	53 (44–59)	47 (42–52)	0.055
Sex male, n (%)	63 (42.3)	23 (67.7)	12 (22.2)	< 0.001
BMI, kg/m^2^ (IQR)	18.2 (16.1–21.7)	22.2 (18.7–25.4)	17.7 (15.2–20.7)	< 0.001
LTx indication, n (%)				N/A
- Fibrosis	42 (28.2)	34 (100)	0	
- Obstructive	55 (36.9)	0	46 (85.2)	
- Vascular	30 (20.1)	0	0	
- Suppurative	14 (9.4)	0	0	
- Allogenic	8 (5.4)	0	8 (14.8)	
Right-side SLTx, n (%)	44 (29.5)	13 (38.2)	31 (57.4)	0.125
Ischemic time, min (IQR)	498 (436–652)	449 (392–497)	457 (427–500)	0.324
Mechanical ventilation, day (IQR)	10 (3–26)	4 (2–13)	4 (2–12)	0.569
Volume reduction, n (%)	28 (18.8)	2 (5.9)	2 (3.7)	0.638
Delayed chest closure, n (%)	40 (26.9)	1 (2.9)	3 (5.56)	0.999
Tracheostomy, n (%)	67 (45.0)	8 (23.5)	13 (24.1)	0.999
ICU stay, day (IQR)	16 (8–33)	9 (6–19)	10 (6–20)	0.658
Three-month mortality, n (%)	10 (6.7)	1 (3.9)	2 (3.7)	0.999

BMI: body mass index; FVC: forced vital capacity; ICU: intensive care unit; ILD: interstitial lung disease; IQR: interquartile range; N/A: not applicable; SLTx: single-lung transplant

### Analysis of native-lung volume, radiological changes, and perfusion on recipients who had undergone single-lung transplantation for ILD

**Fig. 2:**
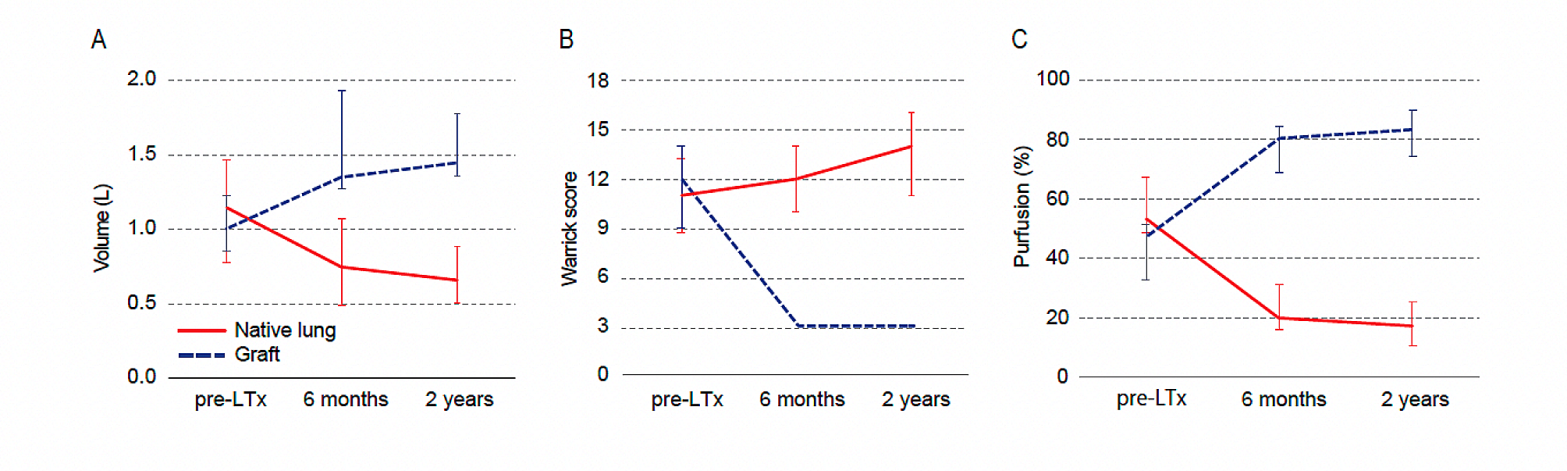
Longitudinal alterations in native lung following single-lung transplantation. The longitudinal changes in the native lung following single-lung transplantation (SLTx) are shown, including (**A**) the trajectory of lung volume, assessed through three-dimensional lung reconstructions, (**B**) radiological alterations using the Warrick Score, and (**C**) perfusion measured by scintigraphy, at three specific time points: before transplantation (pre-SLTx), six months post-SLTx, and two years post-SLTx in recipients who had undergone SLTx for interstitial lung disease

A review of the trajectory of lung volume, radiological alterations, and perfusion in the native lungs of SLTx recipients uncovers the following trends. The median native-lung volume measured 1.145 L (IQR 0.773–1.459) pre-SLTx, which dropped to 0.747 L (IQR 0.489–1.072) at six months post-SLTx, and further declined to 0.653 L (IQR 0.501–0.887) at two years post-SLTx (Fig. 2A). In contrast, the transplanted lung (graft) exhibited a significant increase from 0.996 L (IQR 0.854–1.220) pre-SLTx to 1.354 L (IQR 1.262–1.935) at six months post-SLTx, and 1.435 L (IQR 1.352–1.770) at two years post-SLTx. The Warrick Score, a semi-quantitative scoreing of pulmonary fibrosis on chest CT, revealed a gradual progression of the underlying pulmonary disease over time (Fig. 2B). In the native lung, the score was 11 out of 30 (IQR 8.8–13.3) pre-SLTx, 12 (IQR 10–14) at six months post-SLTx, and 14 (IQR 11–16) at two years post-SLTx. In contrast, minimal radiographic changes were observed in the graft post-SLTx, with a score of 3 out of 30 at both six months and two years post-SLTx. In concordance with the decline in native-lung volume, the ratio of pulmonary perfusion in the native lung exhibited a downward trend (Fig. 2C): 53% (IQR 49–67) pre-SLTx, 20% (IQR 16–31) at six months post-SLTx, and 17% (IQR 11–26) at two years post-SLTx.

### Native-lung complications on recipients who had undergone single-lung transplantation for ILD

**Fig. 3:**
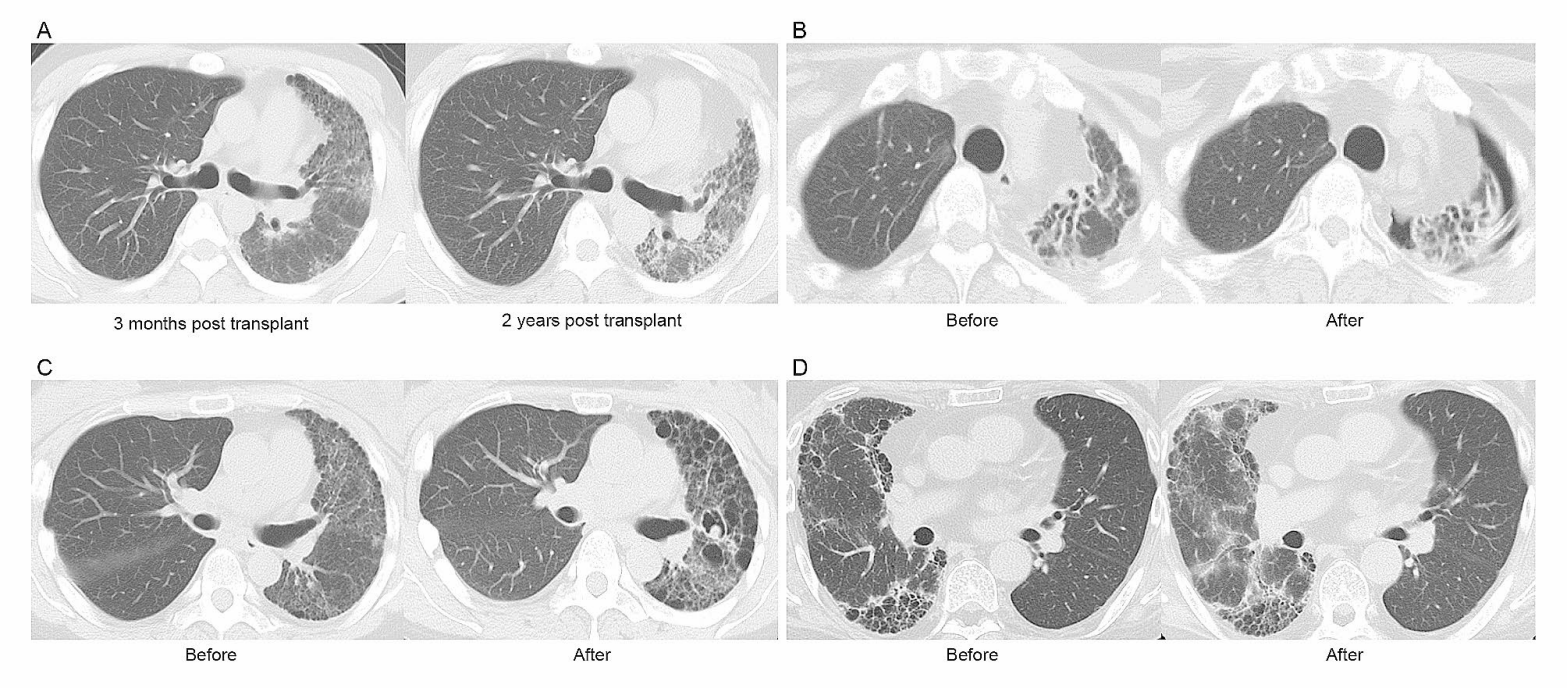
Representative radiological findings. Representative radiological findings are shown, including (**A**) the progression of interstitial lung disease in the recipient’s native lung over a span of three months to two years post-SLTx, (**B**) pneumothorax occurring in the native lung during the acute post-SLTx period, (**C**) pulmonary aspergillosis in the native lung that has developed during the chronic post-SLTx phase, and (**D**) an acute exacerbation of interstitial lung disease in the native lung

Native-lung complications were assessed through a cross-sectional analysis conducted in June 2023 (Table 2). Representative chest CT images of the longitudinal changes and complications in the native lung post-SLTx are presented in Fig. 3. Among recipients who had undergone ILD SLTx, pneumothorax emerged as the most prevalent complication, affecting 10 out of 34 individuals (29.4%). The median time to occurrence was 2.0 months (IQR: 0–46.5), indicating that pneumothorax could manifest both intraoperatively and during the chronic phase post-SLTx. Among these cases, six ILD SLTx recipients required chest-tube insertion, while the remainder were monitored without drainage intervention. Pulmonary aspergillosis in the native lung was identified in four out of 34 (11.8%) ILD SLTx recipients, with a median time to diagnosis of 6.0 months (IQR: 4.8–19.8). Notably, these ILD SLTx recipients developed pulmonary aspergillosis despite receiving antifungal prophylaxis with itraconazole or voriconazole [9]. With the administration of high dosages of voriconazole, no progression of pulmonary aspergillosis was observed. Acute exacerbation of the native lung occurred in three out of 34 (8.9%) ILD SLTx recipients, with a median time to onset of 91.0 months (IQR: 46.0–117.5). All recipients were treated with steroid bolus, resulting in radiological improvement, but they subsequently developed other complications following steroid treatment. Hemoptysis stemming from worsening traction bronchiectasis was observed in three out of 34 (8.9%) recipients, with a median time to onset of 91.0 months (IQR: 46.0–117.5). Two recipients required bronchial artery embolization. Throughout the duration of the study, there were no occurrences of lung cancer or post-transplant lymphoproliferative disorder (PTLD) in ILD SLTx recipients in our center.

**Table 2:** Summary of native-lung complications

Complications in native lung	*n* = 34 (%)	Median Time to events, month (IQR)
Pneumothorax	10 (29.4)	2.0 (0–46.5)
Pulmonary aspergillus	4 (11.8)	6.0 (4.8–19.8)
Acute exacerbation	3 (8.9)	11.0 (7.0–22.5)
Traction bronchiectasis/hemoptysis	3 (8.9)	91.0 (46.0–117.5)
Lung cancer	0	
Post-transplant lymphoproliferative disorder	0	

### Long-term prognosis of recipients who had undergone single-lung transplantation for ILD

**Fig. 4:**
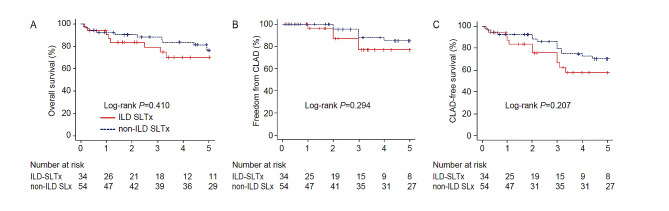
Long-term outcome following single-lung transplantation in interstitial lung disease (ILD) recipients. (**A**) Overall survival was ascertained by considering death from any cause as an event. (**B**) Freedom from chronic lung allograft dysfunction (CLAD) was ascertained by development of CLAD as an event. (**C**) CLAD-free survival was assessed by considering events comprising both the development of CLAD and death from any cause. In cases where no such events occurred, the date of the last follow-up was employed for censoring. The number of recipients at risk was recorded at various time points throughout the duration of the study

ILD SLTx recipients had similar three-month mortality rates to non-ILD STLx recipients (Table 1), prompting a long-term outcome analysis. The outcomes examined included overall survival, freedom from chronic lung allograft dysfunction (CLAD), and CLAD-free survival (Fig. 4). The ILD SLTx recipients exhibited overall survival curves quite similar to those of non-ILD SLTx recipients, although there was a slightly lower survival rate. However, statistical analysis using the log-rank test did not reveal significant differences (*p* = 0.410). In the case of the recipients free from CLAD, significant differences between the two groups were not exhibited (*p* = 0.294). Regarding CLAD-free survival, ILD SLTx recipients also showed a trend of lower survival rates than did non-ILD SLTx recipients, but this difference was not statistically significant (*p* = 0.207). In the context of long-term prognosis, ILD SLTx and non-ILD SLTx recipients yielded comparable results.

## Discussion

While numerous reports have documented post-SLTx complications in the native lung, there is a dearth of studies focusing on their temporal progression, particularly in the context of fibrosis, thus underscoring the unique value of our investigation. This study centers on the analysis of temporal changes and complications in the native lung following ILD SLTx. Elicker et al. previously utilized a two-dimensional approach to assess native-lung capacity in recipients who had undergone SLTx for IPF, revealing a progressive volume loss over time [6]. Conversely, with respect to recipients with systemic sclerosis-related interstitial pneumonia who had undergone SLTx, Hinze et al. categorized diverse chest CT findings into quintiles on the basis of severity and conducted a qualitative analysis concerning fibrosis [7]. Our research introduces a novel semi-quantitative 3D evaluation method to more precisely quantify native-lung volume. Additionally, our investigation encompasses radiological qualitative analysis specifically dedicated to the assessment of lung fibrosis. Furthermore, our study scrutinizes pulmonary perfusion in the native lung following SLTx, revealing a progressive reduction in volume over time, concomitant with the deterioration of radiographic manifestations and a simultaneous decline in perfusion. Our analysis, alongside previous reports, suggests that ILD continues to slowly progress and erode its structural and functional integrity even after SLTx, supporting the notion that native-lung fibrosis continues to deteriorate despite high dosages of an immunosuppressive drug post-SLTx. While this is an anticipated phenomenon from a clinical standpoint, our study represents the first scientific endeavor to systematically evaluate and report on this aspect.

While there have been numerous reports of post-SLTx complications in the native lung, our analysis highlights specific concerns. It is evident from our investigation that the native lung is particularly susceptible to pneumothorax during the perioperative period and remains a source of persistent complications, including the development of pulmonary aspergillosis and acute exacerbation. Notably, these complications, unique to SLTx as opposed to BLTx, have raised concerns regarding the potentially greater complication burden associated with ILD SLTx. At times, complications in the native lung have prompted consideration of native-lung total resection, as indicated in past reports [19]. Gonzalez et al. have reported pneumothorax as a complication that can occur in both the early and late phases following ILD SLTx, but they have concluded that it does not significantly impact long-term outcomes [20]. In our analysis, 29.4% of SLTx recipients with ILD experienced pneumothorax, with a median time to onset of 2.0 months (IQR 0–46.5). Importantly, none of these recipients exhibited any adverse impact on graft function, and no cases were fatal because of native-lung pneumothorax.

On the other hand, post-SLTx aspergillus infection constitutes a severe complication. SLTx presents a higher incidence and mortality rates of pulmonary aspergillus infection than BLTx [21]. This susceptibility extends beyond fungal infections and encompasses bacterial infections as well. Factors such as reduced mucociliary clearance, changes in sputum characteristics, and, in some instances, the establishment of chronic bacterial colonization may contribute to the predisposition to infections and their early dissemination [22]. In our analysis, 11.8% of SLTx recipients with ILD were found to have developed pulmonary aspergillus infection in a native lung. While no fatalities were attributed to this infection, all cases necessitate life-long prophylaxis for the management of the native lung.

Post-SLTx recipients are reliant on graft lung function, and even when complications occur in the native lung, symptoms are often minimal, making acute exacerbations in the native lung easy to overlook. Goletto et al. reported cases of acute exacerbation in the native lung following infection with CMV and SARS-CoV-2 in two SLTx for IPF [23]. Despite progressive fibrosis in the native lung, infection in the graft improved, and the recipients were discharged safely. Robert et al. also observed acute exacerbations in native lungs following CMV infection in three SLTx recipients for IPF, where the chest imaging revealed progressive fibrosis of the native lung with relative sparing of the allograft and the recipients survived [24]. Our three cases of acute exacerbation were not related to infection but occurred when the overall condition of the recipients was compromised. Treatment interventions with steroids bolus were implemented, resulting in one recipient developing severe gastrointestinal complications, while another struggled with steroid-related adverse events due to the inability to reduce steroid dosage. Based on previous reports, acute exacerbation of the native lung after ILD SLTx may not be a direct cause of mortality, and it might be reasonable to opt for observation without intervention as long as it does not affect the graft, even when imaging findings rapidly worsen. However, it is a challenging decision to witness a rapid deterioration in imaging and take no action, which may necessitate further discussion. In any case, acute exacerbation of the native lung following ILD SLTx is a significant complication that cannot be ignored. It should be considered as an important complication of the native lung, and accumulating additional reports in the future will help advance our understanding.

Due to a severe donor shortage, the average waiting time in Japan exceeds 900 days, and the waitlist mortality rate surpasses 50%. Additionally, the absence of an allocation system considering disease progression after listing results in varying mortality rates depending on the underlying disorders [3]. Consequently, the waitlist mortality rate is significantly higher for restrictive lung disorders such as ILD, while obstructive lung disorders such as LAM show a significantly lower rate [8]. In Japan, although ILD has the highest number of listings, the transplantation rate is low. On the other hand, LAM, while being a severe condition, exhibits a remarkably low waiting list mortality rate, leading to a higher proportion of patients undergoing LTx. In this study, the disease distribution reveals LAM as the most prevalent with 35 cases, followed by 14 cases of IPF and 11 cases of CTD-ILD. Therefore, analyzing ILD vs. non-ILD in single-LTx might introduce an imbalance due to the inclusion of a large number of LAM cases in the non-ILD group. Consequently, differences in patient characteristics such as age, sex, and BMI may better reflect ILD in ILD SLTx and LAM in Non-ILD SLTx. In terms of long-term prognosis, LTx for LAM demonstrates superior outcomes compared to transplantation for other diseases, as highlighted by studies conducted by Khawar MU et al [25] and Warrior K et al [26]. However, in this study, although no statistically significant difference was observed in the long-term prognosis between ILD SLTx and non-ILD SLTx, the Kaplan-Meier method reveals that overall survival, freedom from CLAD, and CLAD-free survival for ILD SLTx are lower than those for non-ILD SLTx. This is presumed to be influenced by the higher prevalence of LAM cases in the non-ILD SLTx group.

While our study offers valuable insights into complications of the native lung following ILD SLTx, it is essential to recognize several limitations. Firstly, this research is based on a single-center retrospective analysis, potentially introducing selection bias and limiting the generalizability of our findings. Moreover, the relatively small sample size may impact the statistical robustness of our results. The limited number of events in the native lung further complicated the possibility of drawing definitive conclusions. Additionally, the analysis of SLTx versus BLTx for ILD was hindered by the small subset of ILD recipients (only eight) who had undergone BLTx, making a comparative assessment unfeasible. To enhance our understanding, reliance on existing literature was necessary to explore the prognosis of ILD recipients who had undergone LTx. Furthermore, it is important to acknowledge that the management and outcomes of complications can be influenced by various factors, including individual recipient characteristics and evolving medical practices. Lastly, our study did not delve into the potential impact of varying immunosuppressive regimens, which could be a significant factor in the development of complications.

## Conclusions

Our study sheds light on the unique challenges and complications that persist in the native lung following ILD SLTx. Despite the limitations, our findings contribute to a growing body of knowledge in the field, highlighting the need for ongoing oversight and tailored management strategies to mitigate the impact of these complications following ILD SLTx.

### Electronic supplementary material

Below is the link to the electronic supplementary material.

Supplementary Material 1

## Data Availability

The datasets used and/or analysed during the current study are available from the corresponding author on reasonable request.

## Abbreviations

FVC: Forced vital capacity
ICU: Intensive care unit
ILD: Interstitial lung disease
IQR: Interquartile range
SLTx: Single-lung transplant
