# Supplementary material for: Native-lung complications following single-lung transplantation for interstitial lung disease: an in-depth analysis
Source: BMC Pulm Med. 2024 Apr 24;24:202. doi: 10.1186/s12890-024-03009-6 (PMC11044553; doi:10.1186/s12890-024-03009-6)
Supplement: Supplementary file 1 — Supplementary Material 1 [file 12890_2024_3009_MOESM1_ESM.docx]

Native-Lung Complications Following Single-Lung Transplantation for Interstitial Lung Disease: An In-depth Analysis

Toshikazu Watanabe, Takashi Hirama, Ken Onodera, Hirotsugu Notsuda, Hisashi Oishi, Hiromichi Niikawa, Kazuyoshi Imaizumi, Yoshinori Okada,

**Supplemental table 1. Characteristics of patients who underwent single lung transplant for ILD (n=34) or other than ILD (n=54) and bilateral lung transplant for ILD (n=8)**

| Characteristics | Total  n=149 | ILD SLTx  n=34 | non-ILD SLTx  n=54 | p-value | ILD BLT  n=8 | p-value |
| --- | --- | --- | --- | --- | --- | --- |
| Age, years (IQR) | 46 (36-53) | 53 (44-59) | 47 (42-52) | 0.055 | 37 (25-54) | 0.030 |
| Sex male, n (%) | 63 (42.3) | 23 (67.7) | 12 (22.2) | <0.001 | 3 (37.5) | 0.223 |
| BMI, kg/m^2^ (IQR) | 18.2 (16.1-21.7) | 22.2 (18.7-25.4) | 17.7 (15.2-20.7) | <0.001 | 15.3 (13.2-16.6) | 0.001 |
| LTx indication, n (%) |  |  |  | N/A |  | N/A |
| - Fibrosis | 42 (28.2) | 34 (100) | 0 |  | 8 (100) |  |
| - Obstructive | 55 (36.9) | 0 | 46 (85.2) |  | 0 |  |
| - Vascular | 30 (20.1) | 0 | 0 |  | 0 |  |
| - Suppurative | 14 (9.4) | 0 | 0 |  | 0 |  |
| - Allogenic | 8 (5.4) | 0 | 8 (14.8) |  | 0 |  |
| Right LTx, n (%) | 44 (29.5) | 13 (38.2) | 31 (57.4) | 0.125 |  | N/A |
| Ischemic time, min (IQR) | 498 (436-652) | 449 (392-497) | 457 (427-500) | 0.324 | 618 (442-726) | 0.016 |
| Mechanical ventilation, day (IQR) | 10 (3-26) | 4 (2-13) | 4 (2-12) | 0.569 | 13 (12-15) | 0.033 |
| Volume reduction, n (%) | 28 (18.8) | 2 (5.9) | 2 (3.7) | 0.638 | 5 (62.5) | 0.001 |
| Delayed chest closure, n (%) | 40 (26.9) | 1 (2.9) | 3 (5.56) | 0.999 | 7 (87.5) | <0.001 |
| Tracheostomy, n (%) | 67 (45.0) | 8 (23.5) | 13 (24.1) | 0.999 | 4 (50) | 0.195 |
| ICU stay, day (IQR) | 16 (8-33) | 9 (6-19) | 10 (6-20) | 0.658 | 17 (16-26) | 0.092 |
| Three-month mortality, n (%) | 10 (6.7) | 1 (3.9) | 2 (3.7) | 0.999 | 2 (25) | 0.088 |

BMI, body mass index; FVC, forced vital capacity; ICU, intensive care unit; ILD, interstitial lung disease; IQR, interquartile range; LTx, lung transplant; N/A, not applicable; SLTx, single lung transplant: BLTx, bilateral lung transplant

During the study period, 42 patients underwent deceased-donor lung transplantation (LTx) for ILD, comprising 34 single-lung transplants (SLTx) and 8 bilateral lung transplants (BLTx). The clinical characteristics are detailed in supplemental table 1. There is a tendency for ILD SLTx recipients to be older, with a relatively higher proportion of males, although no statistically significant difference is observed. Ischemic time and days on mechanical ventilation are longer in BLTx, and cases requiring volume reduction surgery and delayed chest closure are more prevalent in BLTx. However, considering the distinction between performing surgery on a single lung and both lungs, the observed differences are not surprisingly substantial.
